# Post-Diagnostic Support and Occupational Therapy Program for Community-Based Dementia Services

**DOI:** 10.1093/geroni/igaa057.2764

**Published:** 2020-12-16

**Authors:** Stephani Shivers, Julie Robison, Erica DeFrancesco, Caroline Kate Keefe, Deidre Sommerer, Christine Bailey, Alis Ohlheiser

**Affiliations:** 1 LiveWell Alliance, Plantsville, Connecticut, United States; 2 University of Connecticut, Farmington, Connecticut, United States; 3 LiveWell Dementia Specialists, Plantsville, Connecticut, United States; 4 Center on Aging | UConn Health, Farmington, Connecticut, United States; 5 UConn Health, Farmington, Connecticut, United States

## Abstract

Service gaps and the absence of a clear-cut care/symptom management pathway for people recently diagnosed with dementia and their family carepartners motivated LiveWell Dementia Specialists to implement a multi-service post diagnostic support program including three occupational therapy (OT) interventions. Program services include an education series on ‘Resilient Living with Dementia’, family coaching and topical education sessions, and OT services including Care of Persons with Dementia in their Environments (COPE), Skills2Care®, and Home Based Memory Rehabilitation. Program services promote adoption of adaptive strategies and action steps to increase carepartner capacity and enhance quality of life among people with dementia. Participants complete assessments at baseline, program completion, and 4- and/or 10-month follow-up. Carepartners show improvements in dementia knowledge (mean baseline score = 24.6, 4-month = 26.0) and preparedness for caregiving (mean baseline score = 18.1, 4-month = 21.9). Program elements and adaptations of COPE for real world practice are discussed.

